# The atlas of variation in healthcare Brazil: remarkable findings from a middle-income country

**DOI:** 10.1007/s43999-022-00017-z

**Published:** 2023-02-08

**Authors:** Henrique Diegoli, Marcia Makdisse, Pedro Magalhães, Muir Gray

**Affiliations:** 1 Academia VBHC Educacao e Consultoria Ltda, São Paulo, Brazil; 2 Value-Based Healthcare Programme, University of Oxford, Oxford, UK

**Keywords:** Regional variation in healthcare, Health inequities, Unwarranted variation, Brazil

## Abstract

**Background:**

Brazil’s Universal Health System is the world’s largest and covers every citizen without out-of-pocket costs. Nonetheless, healthcare inequities across regions have never been systematically evaluated.

**Methods:**

We used government databases to compare healthcare resource utilization, outcomes, expenditure, and years of life lost between 2016 and 2019. The maps used patients’ residences as reference and adjusted for age and private health insurance coverage.

**Results:**

The Atlas shows that for several comparisons, there were no procedures in some regions, including primary coronary angioplasty, thrombolysis for stroke, bariatric surgery, and kidney transplant. Colonoscopy varied 1481.2-fold, asthma hospitalizations varied 257.5-fold, and mammograms varied 133.9-fold. Cesarean births ranged from 19.5% to 84.0%, and myocardial infarction and stroke case-fatalities were 1.1% to 33.7% and 5.0% to 39.0%, respectively. Higher private health insurance coverage in each region was associated with increased resource utilization in the public system in most comparisons.

**Conclusion:**

These findings demonstrate that the SUS does not fulfill the Brazilian constitutional rights due to underutilization, overutilization, and access disparities. The Atlas outlines multiple opportunities to generate value in the SUS.

## Introduction

Variation in healthcare delivery has been the rule, not the exception, across different geographic regions. Unwarranted variation, defined as variation in care delivery that cannot be explained by differences in patient preferences or population needs, is known to have an impact on access to care, health outcomes, and resource allocation [1].

The Darmouth Atlas of Variation was published for the first time in 1996, as a result of extensive population-based research conducted by Professor Jack Wennberg on regional and hospital variation in resource utilization, quality, and cost. It revealed that much of the observed variation in care delivery across regions was unwarranted and frequently reflected physician preferences and local resource availability, and that higher spending and resource use were not associated with better outcomes [2]. Since then, a number of atlases based on this seminal work have been published in developed countries, accelerating the understanding of the causes of variation and contributing to advancements in healthcare [3].

Brazil is a middle-income country with high inequality (Gross Domestic Product per capita [GDP per capita] of US$ 7,518.8 in 2021, and Gini index of 48.9 in 2020) [4, 5], but inequity in healthcare remained unrevealed until the creation of the “Atlas of Variation in Healthcare: Brazil”. The Atlas, released in April 2022 at the Wennberg International Collaborative & Swiss National Science Foundation Spring Synthesis Conference 2022, was created in collaboration with the Oxford Value and Stewardship Programme, under the leadership of Sir Muir Gray. This is the first Atlas of Variation to be published in a middle-income country, as part of the Wennberg International Collaborative [6].

Brazil is the fifth-largest country in the world and the largest in Latin America, with a population of more than 212 million people who share a diverse culture. It is subdivided into five regions (North, Center-West, Northeast, Southeast, and South), 26 states, and one federal district. These states contain a total of 5,570 municipalities.

The Brazilian Healthcare System is comprised of a public sector, funded by taxes and social contributions (SUS, “Unified Health System,” the universal public health system), and a private sector. According to the Brazilian Constitution, created in 1988, “Health is a right of all and a duty of the State and shall be guaranteed by means of social and economic policies aimed at reducing the risk of illness and other hazards and at the universal and equal access to actions and services for its promotion, protection and recovery” [7]. The Brazilian public health system provides care without out-of-pocket payments, entitling all inhabitatns to universal access.

Nonetheless, a considerable proportion of Brazilians are privately insured. Private insurance plays a duplicative role, as it covers healthcare needs that should be met by the public system. The private sector it is partially subsidized by public funding as health costs are tax-deductible [8]. Between 2016 and 2019, the average private health insurance coverage in Brazil was 24.1%, ranging from 5.6% to 40.9% across Health Regions [9].

Descentralization is one of the SUS’s guiding principles, with local governments managing primary care [7]. Regional agreements involving municipalities and Federative Units manage higher complexity healthcare [10]. Since 2011, those agreements are organized in Health Regions, defined as ‘the continuous geographic space constituted by a grouping of neighboring municipalities, delimited from cultural, economic and social identities and communication networks and shared transport infrastructure, with the purpose of integrating the organization, planning’ [11].

The purpose of the “Atlas of Variation in Healthcare: Brazil” is to investigate the degree of variation in the use of health services, outcomes, and costs across the various Brazilian Health Regions in the context of the SUS. This article aims to present the key findings and implications of the Atlas, and offer insights applicable to forthcoming Atlases of Variations in middle-income countries.

## Methods

The “Atlas of Variation: Brazil” is a descriptive study that investigates differences in healthcare resource use, costs, and outcomes across the 450 Health Regions in Brazil. The conditions were selected after ranking all causes of death, procedures, and hospitalizations in order of frequency. The authors then chose the conditions with the highest societal burden and data availability to select 30 maps.

The analysis period was from January 2016 to December 2019; thus, it excluded the effect of the COVID-19 pandemic on the variation in healthcare and established a reference period for future analyses.

All analyses were conducted using the individual’s place of residence as the point of reference. For instance, if a resident of one state performed surgery in another, the surgery rate in the patient’s home state would increase. This method was used to improve the evaluation of healthcare for populations traveling between locations to receive medical care.

The analyses were carried out at two different levels: the Federative Unit and the Health Regions. The Federative Units are the 26 states and the Federal District. The Health Regions are the primary unit of analysis for each map because they more accurately depict patterns of access and quality of services [11]. In 2019, each Health Region had an average population of 461,446 people.

Each analysis’ results were presented in maps, tables, and charts. Each Federative Unit was divided into five percentiles on charts and maps using a simple division procedure based on the number of observations. The percentiles were shown in varying shades, with darker representing a greater frequency or proportion and lighter representing a lesser frequency or proportion. All analyses were conducted in Microsoft Excel 365®. In each graph, the columns represented the observed measure in each state, while the gray circles represented the observed measure in each health region of the corresponding state.

The indirect method was used for age standardization, which calculates the expected rate for each region based on the observed rate in Brazil and divides it by the observed rate to obtain the standardized rate. In the maps that investigate the frequency of resource use (rates), an additional analysis was performed in which the population at risk consisted solely of residents who did not have private health insurance and were thus regarded as exclusive SUS users, with all other calculations remaining unchanged. This analysis seeks to decrease the bias created by the different coverage by health insurance locations in Brazil, given that most health insurance beneficiaries receive care outside of the SUS. This prevents an underestimation of the usage of SUS resources in areas with substantial health plan coverage.

### Data source

Regarding the availability of digital health data, the Brazilian Ministry of Health has registered at least 146 health and healthcare databases as of the present [12]. These databases contain raw data from various sources and formats, some covering the entire country of Brazil and others only specific services.

These databases are aggregated in the DATASUS, an information system that includes data from all providers that treat SUS patients, and some data from services outside the SUS. Data collection was conducted utilizing official data from several information systems linked to the DATASUS [13]. The following databases were utilized to extract data:

- Mortality Information System (SIM), containing information from death certificates issued throughout the national territory, such as age, cause of death, and location of death, among other information. It contains information from all death certificates, without distinction between SUS and private health insurance holders. Consequently, death and lost years of life charts included the entire population, not just SUS users.
- Outpatient Information System (SIA) is the information system containing data pertaining to outpatient procedures performed in the SUS, including medication requests, exams, and consultations, among others. Authorizations for Outpatient Treatments (APAC), documents used to obtain medication and other high-cost procedures, served as the major data source for the maps of outpatient resource utilization. The SIA includes all SUS providers, but does not cover privately insured procedures.
- Hospital Information System (SIH), which includes hospital admissions in the SUS and provides data on diagnosis, procedures performed, length of hospital stay, and percentage of deaths. The SIH also covers all hospitals in the SUS but does not cover patients or hosptials in the private sector.
- The Brazilian Institute of Geography and Statistics (IBGE) provided demographic information about the population of each Health Region and State.

## Results

In 30 distinct maps, healthcare variation was compared for diseases of 11 medical specialties: cardio-cardiovascular diseases (9), oncological diseases (5), respiratory diseases (5), metabolic diseases (2), gastrointestinal diseases (2), musculoskeletal diseases (2), neurological diseases (1), ophtalmic diseases (1), kidney diseases (1), pregnancy, labor and delivery (1), and mental disorders (1). The healthcare conditions were compared utilizing rates of hospitalizations (8), rates of surgical procedures (7), years of life lost for disease groups (2), diagnostic tests (2), the proportion of admissions utilizing specific treatments (2), case-fatality (2), length of hospital stay (2), total expenditure (1), outpatient prescription pattern (1), diagnostic test appropriateness (1), the proportion of deaths at home (1), and the proportion of cesarean births (1).

Between 2016 and 2019, some Health Regions did not document any procedure or hospitalization related to specific conditions, implying that the population did not have access to these services. These include thrombolysis for stroke treatment, angioplasty for myocardial infarction, bariatric surgery for obesity, dementia drug prescriptions, cataract surgery, tonsillectomy, ruptured disc or spinal fusion surgery, kidney transplant, and hospitalization for persons at high risk for suicide.

In 270/450 Health Regions (60.0%), home to 77,669,294 (34.0% of the Brazilian population in 2019), no thrombolysis for stroke treatment was offered in the period, and thrombolysis was used to treat less than 1% of stroke patients in 394/450 Health Regions (87.6%), home to 154,759,429 (73.7% of the Brazilian population). Angioplasty for myocardial infarction was not offered to any patient in the period in 51/450 Health Regions (11.3%), home to 9,937,806 (4.7% of the Brazilian population). The case-fatality of myocardial infarction and stroke showed alarmingly large variations among Health Regions (1.1% to 33.7% and 5.0% to 39.0%, respectively).

Bariatric surgery for obesity treatment was not used to treat any patients in 75/450 Health Regions, home to 21.242.138 (10,1% of the Brazilian population). Despite the fact that the median age-adjusted rate of bariatric surgery was 1 per 100,000 people, 5 Health Regions had rates that were higher than 68.5 per 100,000, which may indicate overuse in those areas, underuse in areas with lower rates, or a combination of the two.

The rate of colonoscopy or flexible sigmoidoscopy had the highest variation in the Atlas (0.6 to 880.3 per 100,000 inhabitants per year, 1481.2-fold). The procedure had a median rate of 76.8 per 100,000, with 5 Health Regions demonstrating rates above 634.1, and 5 Health Regions with rates under 4.4.

Hospitalizations for asthma also had a high variation (1.9 to 488.0 per 100,000 inhabitants per year, 257.5-fold), as was observed in the number of mammograms in women aged 50–69 years (140.7 to 18,831.8 per 100,000 women aged 50–69 years per year, 133.9-fold variation).

There was massive variation in the proportion of cesarean births (19.5% to 84.0%), suggesting widespread overutilization. There was substantial variation within each state. The States with wider variation were São Paulo (27.1% - 83.0%), Minas Gerais (26.1% - 77.1%), Paraná (33.9%–79.1%) and Paraíba (38.0% - 81.4%), located in three different Brazilian regions with diverse economic and social backgrounds.

The proportion of cancer deaths at home varied widely (4.1% to 53.6%). Surprisingly, states with high proportions of cancer deaths at home have some of the lowest Gross Domestic Product per capita in Brazil (Ceará, Piauí, Amazonas, Maranhão, Alagoas, Paraíba). Therefore, those high proportions are probably related to insufficient access to hospital care rather than better palliative care services.

The correction by health insurance coverage increased variation in the majority of comparisons due to a more significant increase in the rate in places with already high rates and higher insurance coverage. For instance, the fold-variation for colonoscopy or flexible sigmoidoscopy rose from 1,481.2 to 1,757.3, and the fold-variation for mammograms rose from 133.9 to 150.7.

**Fig. 1:**
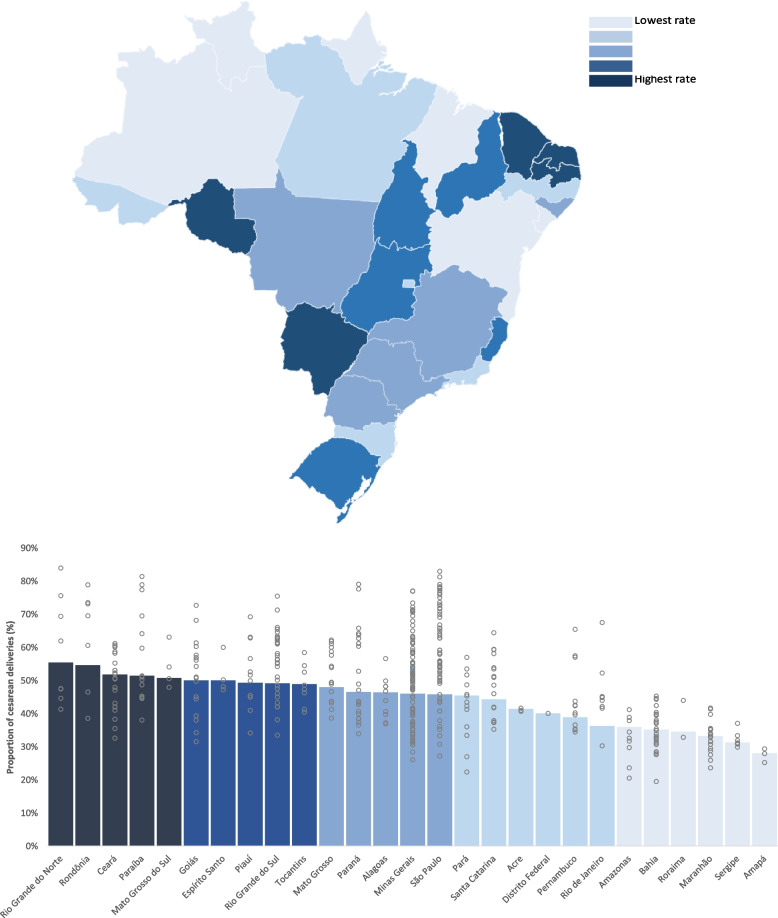
Proportion of Cesarean Deliveries. This figure shows the variation in the proportion of births that are cesarean deliveries in Brazil. Columns indicate the average in each state, while gray circles indicate the average in the regions within each state

**Fig. 2:**
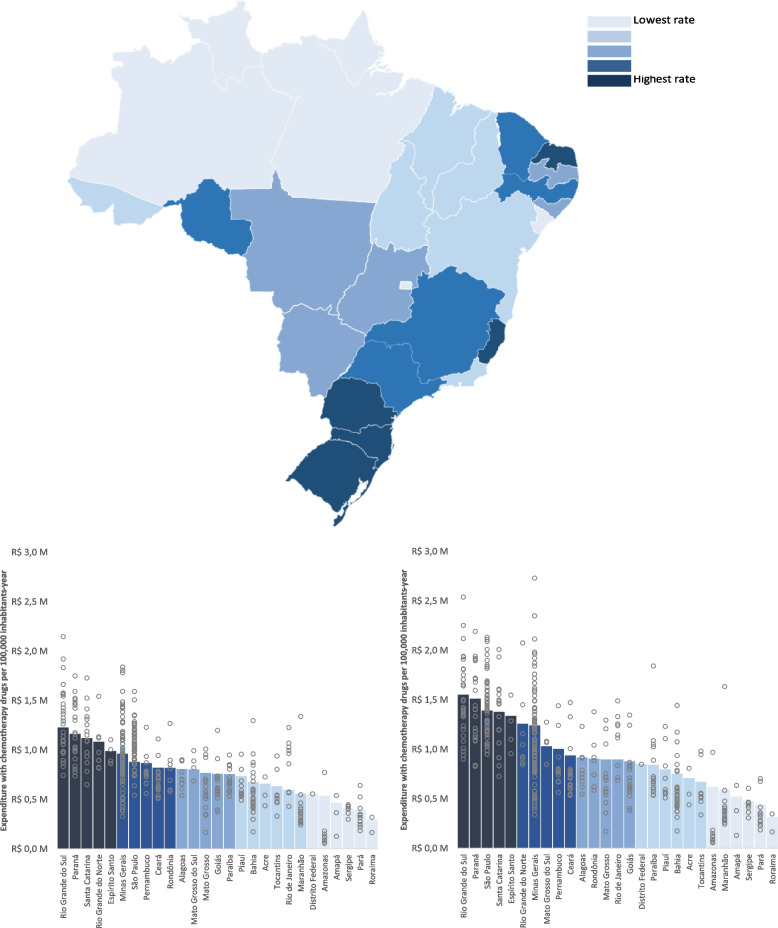
Expenditure in chemotherapy. This figure shows the variation in chemotherapy expenditure across states and health regions in Brazil. Columns indicate the average in each state, while gray circles indicate the average in the regions within each state

**Fig. 3:**
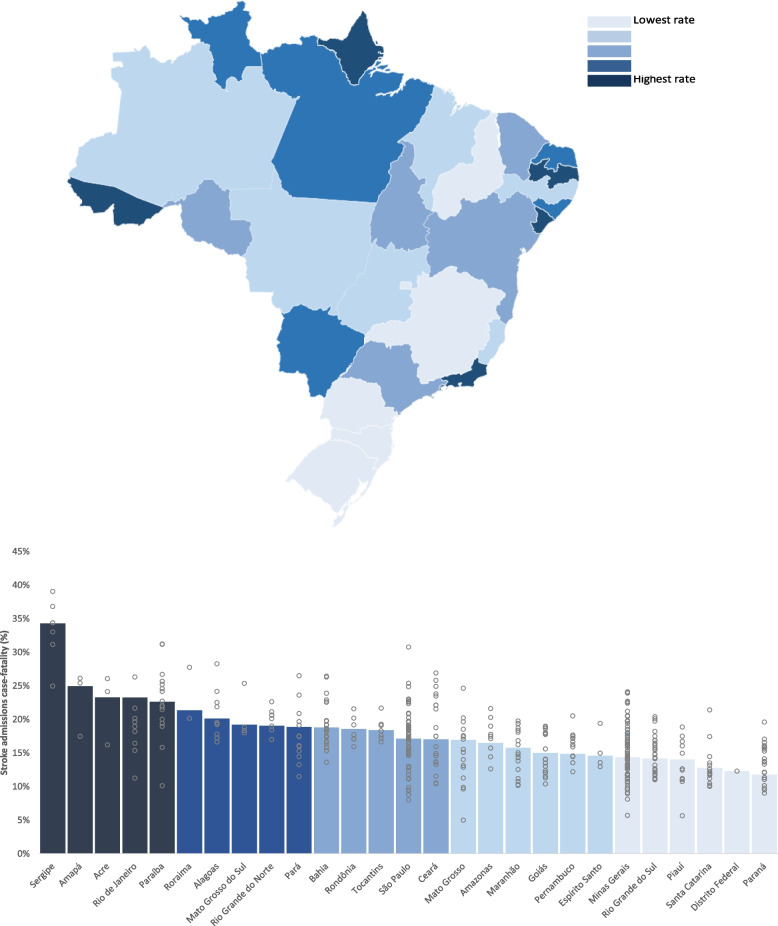
Case-fatality for stroke. This figure shows the variation in case-fatality for stroke admissions in Brazil. Columns indicate the average in each state, while gray circles indicate the average in the regions within each state

The complete Atlas is available for free download in English and Portuguese [14] (Figs. 1, 2 and 3).

## Discussion

This article described the methodology and key findings of the first Atlas of Variation in Healthcare to be compiled in Brazil, one of the first in developing nations. The maps enable comparisons of the patterns of access to services, outcomes, and diverse practices of healthcare professionals across Brazil.

Brazil has the world’s largest universal health-care system, and its fundamental cornerstones are universalization, equity, and integrality (providing healthcare at all levels), as guaranteed by the Brazilian constitution. The Brazilian Atlas uncovered a significant variation in access to and utilization of health-care resources, contrasting with the principles of the SUS (Table 1).

**Table 1:** A summary of the Atlas’s findings

Map title	No adjustment by health insurance coverage		Adjustment by health insurance coverage^**a**^	
	Range of variation	Fold-variation	Range of variation	Fold-variation
Years of life lost due to cardiovascular disease (age-adjusted years lost per 100,000)	320–1677	5.2		
Hospitalizations for heart failure (age-adjusted incidence per 100.000)	26.2–568.7	21.7	25.9–565.7	21.9
Hospitalizations for acute myocardial infarction (age-adjusted incidence per 100.000)	9.1–273.8	30.0	9.1–314.1	34.3
Length of hospital stay for acute myocardial infarction (number of days per admission)	2.2–15.4	7.1		
Case-fatality rate for acute myocardial infarction (% of admissions)	1.1% - 33.7%	32.0		
Primary coronary angioplasty for the treatment of acute myocardial infarction (age-adjusted incidence per 100.000)	Zero - 39.3	N/A	Zero - 46.2	N/A
Hospitalizations for stroke (age-adjusted incidence per 100.000)	34.0–195.4	5.7	39.9–240.1	6.0
Thrombolysis for the treatment of stroke (% of stroke admissions)	Zero - 33.1%	N/A		
Case-fatality rate for stroke (% of admissions)	5.0% - 39.0%	7.9		
Hospitalizations for diabetes (age-adjusted incidence per 100.000)	6.2–237.7	38.3	10.3–273.6	26.7
Bariatric surgery for obesity treatment (age-adjusted incidence per 100.000)	Zero - 108.7	N/A	Zero - 116.4	N/A
Prescription of medication for dementia (age-adjusted incidence per 100.000)	Zero - 2717.8	N/A	Zero - 4.160.1	N/A
Cataract aurgeries (age-adjusted incidence per 100.000)	Zero - 341.9	N/A	Zero - 406.5	N/A
Years of life lost due to early cancer deaths (age-adjusted years lost per 100,000)	231–1317	5.7		
Number of mammograms in women aged 50 to 69 years (age-adjusted incidence per 100,000)	140.7–18,831.8	133.9	144,1 - 21,708.7	150.7
Proportion of women aged 50 to 69 years who underwent a mammogram (%)	35.1% - 96.2%	2.7		
Proportion of cancer deaths at home (%)	4.1% - 53.6%	13.1		
Expenditure on chemotherapy drugs (age-adjusted, in 2019 Brazilian Reais)	54,919 - 2,146,526	39.1	54,966 - 2,729,017	49.7
Surgical treatment of femoral neck and hip fractures (age-adjusted incidence per 100,000)	3.1–74.9	23.8	2.0–80.0	40.5
Spine Surgery: Herniated disc surgery or spinal fusion/arthrodesis (age-adjusted incidence per 100,000)	Zero - 43.8	N/A	Zero - 53.6	N/A
Kidney Transplant (age-adjusted incidence per 100,000)	Zero - 6.1	N/A	Zero - 9.8	N/A
Colonoscopy or flexible sigmoidoscopy (age-adjusted incidence per 100,000)	0.6–880.3	1481.2	0.6–1056.9	1.757.3
Inguinal hernia repair/hernio-plasty (age-adjusted incidence per 100,000)	21.2–138.1	6.5	22.2–178.9	8.1
Proportion of cesarean deliveries (% of all births)	19.5% - 84.0%	4.3		
Tonsillectomy and/or adenoidectomy (age-adjusted incidence per 100,000 between 0 and 19 years old)	Zero - 611.2	N/A		
Hospitalizations for pneumonia or influenza (age-adjusted incidence per 100,000)	81.9–1201.5	14.7	82.1–1209.3	14.7
Length of hospital stay for pneumonia or influenza (number of days per hospital admissions)	3.0–9.7	3.3		
Hospitalizations for Asthma (age-adjusted incidence per 100,000)	1.9–488.0	257.5	2.3–580.9	249.1
Hospitalizations for emphysema and other chronic obstruc-tive pulmonary diseases (age-adjusted incidence per 100,000)	3.5–407.4	116.6	5.2–416.9	80.6
Hospitalizations of people at high risk of suicide (age-adjusted incidence per 100,000)	Zero - 209.5	N/A	Zero - 265.5	N/A

^a^The only metrics adjusted for health insurance coverage were rates; therefore, other metrics are left blank

Multiple atlases have been created to investigate unwarranted variation in other countries, and they have been used to tackle underutilization or overutilization of healthcare [3]. The Brazilian Atlas has some of the highest variations ever documented, which is illustrative of the challenges faced by developing and highly unequal nations. Substantial variation was seen across all geographic regions. The maps show evidence of healthcare resource underutilization, overutilization, and inequitable access in the SUS. The magnitude of variations observed in Brazil reveal significant opportunities to increase value for the Brazilian population.

As an illustration of overutilization, it is noted that the proportion of cesarean deliveries in certain Health Regions in the SUS exceeded 80%, well above the 10–15% rates recommended by the World Health Organization [15]. None of the health regions had values within this recommended range, implying significant overutilization.

On the other hand, thrombolysis for stroke and angioplasty for myocardial infarction were underutilized in most places. Data on the availability of other treatments, such as stroke units and thrombectomy for stroke and thrombolysis and coronary unit for myocardial infarction, were sought but were unavailable. Nonetheless, the available data indicate a significant underutilization of best practices in the treatment of stroke and myocardial infarction, which are leading causes of death and disability in Brazil.

The Atlas showed considerable underutilization of many programs recommended by the Ministry of Health. In certain Federative Units and Health Regions, the frequency of operations such as thrombolysis for stroke, primary angioplasty for myocardial infarction, and kidney transplant was zero, meaning that no residents in the SUS had access to these services. For other comparisons, such as colonoscopies or mammograms, the rate was not zero, but it appears to be significantly lower than what clinical recommendations would consider reasonable.

The evaluation at the Health Region level allowed us to identify significant findings that would not have been apparent if only the Federative Units were directly compared. Often, the variation within each Federative Unit was greater than the variation across states, highlighting the necessity of exploring variations in the quality and availability of services within each state’s Health Regions.

The standardization of rates by age allowed for a direct comparison among different locations, minimizing age group disparities that could influence the utilization of health resources. A correction was made by health insurance coverage in an effort to eliminate unwarranted variation. On the majority of maps, however, there was an increase in variation following rectification by health plan coverage. Despite the fact that people with private health insurance can also use the SUS, this finding suggests that SUS users in locations with high coverage by health insurance utilize more SUS resources than those in regions with low coverage. This difference may have resulted from the greater availability of health services in those places, which may be related to supply-sensitive care.

### Lessons learned

#### A vast quantity of digital health data is accessible and awaiting transformation into useful knowledge

We’ve heard repeatedly that Brazil lacks sufficient data to guide resource allocation and reduce health care disparities. We demonstrate that the 146 public health and healthcare databases in our nation, which contain open and unprocessed data from different sources and formats and can still be further explored, are not being used to their full potential. Even though data on care effectiveness and health outcomes are scarce, the available data on variation in care delivery is sufficient to start a movement that will lead to further investigations on the causes and opportunities for value improvement.

#### Collaboration is key

Brazil’s Atlas of Variation was created with the Oxford Value and Stewardship Programme and the Wennberg International Collaborative. Their generosity in sharing their experiences and insights enabled us to build the Atlas.

#### Highlight variation at the level where decisions regarding funding allocation are made

The analysis of variation at the level of Health Regions revealed significant findings that were not visible across Federative Units and provided a more accurate depiction of patterns of access to and quality of health services. Frequently, variation within each Federative Unit was greater than variation between them. As Health Regions are the reference point for resource transfers across the Federative entities, highlighting variation at this level enables local benchmarking and provides public managers with data that can aid in the funding allocation process.

#### Adjusting for health insurance coverage

The Unified Health System (SUS) and a robust private sector make up Brazil’s healthcare system. To reduce the bias on resource utilization caused by insurance coverage and prevent an underestimation of SUS health services, an additional analysis was performed on residents without private health insurance coverage (‘Exclusive SUS beneficiaries’). In spite of this, most maps showed an increase in disparities after accounting for health insurance coverage, demonstrating that areas with more insurance coverage also offer more healthcare in the SUS, thereby amplifying healthcare inequity.

### Limitations

The quality of available data can differ from location to location. Nonetheless, we sought to use the most reliable public sources when compiling the Atlas, which also describes the reimbursement for healthcare in Brazil.

This Atlas serves as a basis for additional debate and analysis. Without more in-depth statistical analysis, it relies on descriptive data to present an overview of the existing variation in healthcare in Brazil.

## Conclusions

The first Atlas of Variance in Healthcare: Brazil presents evidence of high variation in healthcare resource utilization and outcomes in the SUS, which reflects underutilization, overutilization, and inequity. Correction by the prevalence of health insurance did not minimize the variation in the majority of comparisons. The magnitude of variation observed across geographic regions is unlikely to be explained primarily by differences in patient needs and preferences and further analyses is required to identify causes, gaps in care and challenges around care variation.

These results demonstrate that the SUS falls far short of guaranteeing the universal and integral access to healthcare rights set forth in the Brazilian constitution. Opportunities are identified to generate value for the Brazilian population in multiple health conditions.
